# Management of cryptococcal meningitis in a district hospital in KwaZulu-Natal: A clinical audit

**DOI:** 10.4102/phcfm.v6i1.672

**Published:** 2014-10-13

**Authors:** Benjamin O. Adeyemi, Andrew Ross

**Affiliations:** 1 Department of Family Medicine, Pietermaritzburg Hospitals Complex and University of KwaZulu-Natal, South Africa; 2 Department of Family Medicine, University of KwaZulu-Natal, South Africa

## Abstract

**Background:**

Despite the development of context-specific guidelines, cryptococcal meningitis (CCM) remains a leading cause of death amongst HIV-infected patients. Results from clinical audits in routine practice have shown critical gaps in clinicians’ adherence to recommendations regarding the management of CCM.

**Aim:**

The aim of this study was to review the acute management of CCM at an urban district hospital in KwaZulu-Natal, South Africa with a view to making recommendations for improving care.

**Setting:**

An urban district hospital in KwaZulu-Natal, South Africa.

**Methods:**

A retrospective audit was performed on clinical records of all patients (age > 13 years) admitted to the hospital with a diagnosis of CCM between June 2011 and December 2012.

**Results:**

Measurement of cerebrospinal fluid opening pressure at initial lumbar puncture (LP) was done rarely and only 23.4% of patients had therapeutic LPs. The majority of patients (117/127; 92.1%) received amphotericin B, however, only 19 of the 117 patients (16.2%) completed the 14-day treatment target. Amphotericin B-toxicity monitoring and prevention was suboptimal; however, in-patient referral for HIV counselling and testing was excellent.

**Conclusions:**

The quality of care of CCM based on selected process criteria showed gaps in routine care at the hospital despite the availability of context-specific guidelines. An action plan for improving care was developed based on stakeholders’ feedback. A repeat audit should be conducted in the future in order to evaluate the impact of this plan and to ensure that improvements are sustained.

## Abstrait

**Gestion de la méningite cryptococcique dans un hôpital régional au KwaZulu-Natal: une vérification Clinique**.

**Historique:**

Malgré le développement de directives spécifiques au contexte, la méningite cryptococcique (CCM) reste une des causes principales de décès parmi les patients infectés du VIH. Les résultats des vérifications cliniques dans la pratique courante ont montré des lacunes critiques dans l'adhésion des cliniciens aux recommandations sur la gestion du CCM.

**Objectif:**

Le but de cette étude était de revoir la gestion active du CCM dans un hôpital régional urbain du KwaZulu-Natal, en Afrique du Sud afin de faire des recommandations pour améliorer les soins de santé.

**Lieu:**

un hôpital régional urbain au KwaZulu-Natal, en Afrique du Sud.

**Méthodes:**

Les dossiers cliniques de tous les patients (âgés de plus de 13ans) admis à l'hôpital avec un diagnostique de CCM de juin 2011 à décembre 2012 ont été vérifiés rétrospectivement.

**Résultats:**

La mesure de la pression d'ouverture du CSF à la ponction lombaire initiale (PL) a rarement été effectuée et seuls 23.4% des patients ont subi des PL thérapeutiques. La majorité des patients (117/127; 92.1%) ont reçu de l'amphotéricine B. Cependant, seuls 19 sur 117 patients (16.2%) ont atteint l'objectif de traitement de 14 jours. L'identification et la prévention de la toxicité de l'Amphotéricine B laissaient à désirer. Cependant, l'admission des patients en consultation interne pour le conseil et dépistage du VIH était excellente.

**Conclusions:**

La qualité des soins de la CCM basés sur des processus de sélection des critères a révélé des lacunes dans les soins courants à l'hôpital malgré la disponibilité des lignes directrices particulières au contexte. Un plan d'action pour améliorer les soins a été développé basé sur les feed-back des parties prenantes. Il faudra faire dans le futur une vérification de contrôle pour évaluer l'impact de ce plan et assurer que les améliorations sont maintenues.

## Introduction

Cryptococcal meningitis (CCM) has emerged as one of the leading causes of death amongst HIV-infected patients worldwide.^1, 2^ Recent epidemiological shifts and an explosion of the HIV pandemic have resulted in the emergence of CCM as a major public health problem and a leading cause of community-acquired meningitis in sub-Saharan Africa.^3, 4^ Estimates from sub-Saharan Africa in 2009 suggested that CCM killed more people than tuberculosis – with more than half-a-million deaths recorded per annum.^1, 5, 6^

HIV-associated CCM is a life-threatening disease with a 100% case fatality rate in the absence of treatment.^7^ Effective management of CCM requires rapid fungicidal induction therapy with amphotericin B and flucytocine, followed by suppressive therapy with fluconazole, as well as aggressive management of raised intracranial pressure (ICP) and appropriate management of immune reconstitution inflammatory syndrome (IRIS).^4, 6, 8^ Raised ICP, which often complicates CCM,^4, 9^ is a common cause of severe headache, neurological deficit and death.^9^

Despite restricted access to flucytocine, there are effective guidelines for the management of HIV-associated CCM in South Africa.^10^ The South African treatment guidelines for the management of CCM comply substantially with best practices^11^ by emphasising the use of amphotericin B for induction, advocating the aggressive management of raised ICP as well as the use of secondary prophylaxis with oral fluconazole in order to prevent recurrence of CCM.^10, 12^ The guidelines also provide recommendations for the prevention and management of amphotericin B-related nephrotoxicity through provision of routine pre-hydration and electrolyte supplementation for patients on amphotericin B.^10–12^ In line with the recognition of the association between improved survival outcomes and timely introduction of antiretroviral therapy (ART),^4, 10, 13^ clinicians are also enjoined to ensure that patients with AIDS-related CCM are initiated on lifelong ART.^10, 12, 14^

Cryptococcal meningitis is a common AIDS-defining illness seen at the hospital where this audit was conducted. An exploratory clinical audit carried out in 2006 on the management of CCM at the hospital highlighted concerns about the management of patients with CCM and drew attention to the unavailability of clearly-defined, local clinical guidelines at that time.^15^ Context-specific, comprehensive and evidence-based guidelines for the management of HIV-associated CCM in South Africa were introduced at the hospital in 2007 following the aforementioned audit.^10, 12^ Other studies have shown that despite the availability of national guidelines there is no evidence to suggest that the availability of these guidelines has been associated with significant corresponding improvements in care of and outcomes for HIV-infected patients with CCM in South Africa.^16, 17^ Critical gaps in routine management of CCM were also highlighted by a clinical audit conducted in the United States.^18^

The aim of this study was to conduct a clinical audit of clinicians’ level of adherence to published context-specific guidelines on the management of CCM with a view to making recommendations for the improvement of care. Whilst some have argued that the evidence for the usefulness of clinical audit on improving professional practice is not particularly strong,^19^ a Cochrane Systematic Review^20^ on the effects of clinical audit and feedback on professional practice and clinical outcomes concluded that audit is an effective tool for improving practice. In fact, the positive effects of clinical audit may be magnified greatly when the feedback emanating from the audit is given in an intense manner,^20^ especially in settings where the initial baseline adherence to practice guidelines is low.

## Research method and design

This was a clinical audit conducted at an urban district hospital in KwaZulu-Natal, South Africa using a retrospective descriptive study design. The hospital has a full package of district-level services.

The clinical audit topic was selected based on routine observation of the high mortality seen amongst patients with CCM at the hospital and the recognition of the possible impact of improved quality of care on mortality outcomes. An audit team, supported by a broader team of healthcare workers, was formed. The study method comprised the four essential steps of a quality improvement cycle: agreeing on criteria and setting target standards; collecting data on current practice; comparing current practice with target standards; and, finally, planning care and implementing changes.^21^

Based on the prevailing published guidelines over the study period,^10–12^ criteria were developed around key issues regarding the diagnosis and management of HIV-associated CCM. The key issues selected for audit were: baseline cerebrospinal fluid (CSF) manometry and management of raised ICP; amphotericin B treatment and prevention and/or management of its toxicity; as well as patients’ linkage with antiretroviral services. Target standards were set based on published literature^15–18^ and with due consideration for the complexity and constraints of the study setting.^21^

The clinical records of all patients aged 13 years and older who were admitted to the hospital with a diagnosis of CCM between 01 June 2011 and 31 December 2012 were reviewed retrospectively in order to extract information regarding clinical data and patient management. A diagnosis of CCM based on a positive India ink, positive cryptococcal latex agglutination test or a positive culture of *Cryptococcus neoformans* was essential for inclusion in this study. Patient records were identified using the hospital's admission, transfer and discharge registers. Further review of clinical records, mortality records and laboratory data (accessed from the National Health Laboratory Service [NHLS] database) was done in order to extract information on the patients’ demographic data and parameters relevant to the audit criteria. A data extraction tool was developed to collect the information and the data were captured onto a Microsoft^®^ Excel spreadsheet. Simple frequencies and percentages were generated for agreed criteria and these were compared to target standards.

The results of the clinical audit were presented at an audit feedback meeting at the hospital in November 2013. The meeting was attended by senior management staff, NHLS laboratory staff representatives, pharmacy representatives, stores department staff, medical doctors, nursing staff and other members of the healthcare team involved in caring for medical patients. The gathering reflected on the findings and the implications of the identified practice gaps. An action plan aimed at improving the quality of acute management of HIV-associated CCM was agreed upon following extensive discussions amongst the stakeholders present at the meeting.

### Ethical considerations

Ethical approval was obtained from the University of KwaZulu-Natal Biomedical Research Ethics Committee (REF: BE086/12) and the management of the hospital.

## Results

The demographic and clinical profiles of the patients included in the study are presented in Table 1.

**TABLE 1:** Demographic and clinical profiles of patients admitted with cryptocccal meningitis.

Variable	Male	Female
No. of patients (%)	65 (51.2)	62 (48.8)
Age (yrs) median (IQR)	37 (31–42)	33 (27–42)
CD4 cell count (cells/mm^3^)† - Median (IQR)	36 (19–80)	56 (23–137)
CCM Case definition - New	49	53
Symptomatic relapse	16	9

IQR, interquartile range

† CD4 count results available for 74 patients only

CCM, cryptococcal meningitis.

Table 2 shows the criteria selected for audit and the level of performance compared to target standards for each criterion. Of the 95 patients who had no therapeutic lumbar puncture (LP), there was no evidence in the notes that the procedure was offered to 82 (86.3%) of the patients; and it was declined by 2 (2.1%) patients and contraindicated in one (1.1%) patient who needed further assessment for new-onset blindness. In 8 cases (8.4%), the patients died before the LP was performed, even though the clinician(s) had made a decision to do a therapeutic LP. There was one instance (*n* = 1; 1.1%) where the clinical record showed that there was no manometer and another where many LP attempts were recorded as ‘unsuccessful’ (*n* = 1; 1.1%).

**TABLE 2:** Comparison of performance and target standards.

Criteria	Target standard (%)	Number of patients (*n*)	Performance standard *n* (%)	Target met (yes/no)
CSF manometry at initial LP	80	127	2 (1.7)	No
ICP monitoring and therapeutic LP	100	124†	29 (23.4)	No
Weight-based correct dosing of amphotericin B	80	117‡	23 (19.7)	No
Complete 14-day amphotericin B	80	117‡	19 (16.2)	No
Saline loading	100	117‡	68 (58.1)	No
Prophylactic magnesium supplementation	100	117‡	5 (4.3)	No
Prophylactic potassium supplementation (unless contraindicated)	100	117‡	31 (26.5)	No
Adequate renal function monitoring (3x/ week U&E, Mg)	100	80†	26 (32.5)	No
Weekly haemoglobin monitoring	100	72†	14 (19.4)	No
Referral for HIV counselling and testing when admission HIV status was unknown and/or negative	100	31†	31 (100)	Yes
Incidence of renal impairment	< 40%	88†	32 (36.4)	Yes
Two-week mortality	< 40%	127	71 (55.9)	No

CSF, cerebrospinal fluid; LP, lumbar puncture; ICP, intracranial pressure; U&E, urea and electrolytes; Mg, magnesium

† Total number of patients < 127 because of missing data and early mortality making evaluation of adequacy of monitoring for toxicity impossible

‡ Only 117 patients received amphotericin B.

Out of the 127 patients, analgesics (prescribed separately and in fixed-dose formulations) were employed for the management of headaches associated with CCM in 104 (81.9%) of the patients in this study. Seventy-seven (60.6%) patients had paracetamol; 58 (45.7%) had a paracetamol-codeine fixed-dose combination analgesic; 27 (21.3%) had non-steroidal anti-inflammatory drugs whilst 15 (11.8%) had other opiates such as tramadol and morphine.

Assessment of the pattern of amphotericin B prescription showed that the most frequently-prescribed daily dose was 50 mg. Evidence of determination of actual dose using patients’ weight was found for only 23/127 (18.1%) patients. The median duration of induction therapy with amphotericin B was 8 days (interquartile range [IQR] 3–5 days). Only 19/127 (15.4%) patients completed 14 days of amphotericin B. The reasons why patients did not complete 14 days of amphotericin B were as shown in Table 3.

**TABLE 3:** Reasons for amphotericin B induction therapy < 14 days.

Reason	Frequency (*n* = 104)	%
Died	61	58.7
Improved	23	22.1
Toxicity	10	9.6
Not prescribed	4	3.9
Renal failure at diagnosis	3	2.9
Refusal of hospital treatment	2	1.9
Out of stock (amphotericin B)	1	1.0

## Discussion

Results of this clinical audit from routine practice in a district hospital provide evidence of important gaps in the clinical management of CCM. Similar to findings from a clinical audit conducted in Washington D.C. amongst patients treated for CCM,^18^ failure to measure CSF opening pressure at initial LP was the most common deviation from clinical guidelines in our setting. The percentage of patients who had their CSF opening pressure measured at baseline in this audit was 10 times lower than figures (1.7% versus 17%) reported amongst patients seen at the district hospital level in the Western Cape.^17^ Clinicians’ failure to perform routine measurement of CSF opening pressure at baseline has been attributed to non-availability of spinal manometers;^17^ clinicians’ limited awareness of the epidemiological significance of *C. neoformans* as one of the leading causes of community-acquired meningitis; and inadequate knowledge of important role of ICP management in the treatment of CCM.^18^ The consensus from discussions at the audit feedback meeting was that spinal manometers were generally available in the hospital. This assertion was supported by the fact that there was only one instance where the clinical record showed that the CSF opening pressure was not measured because of the non-availability of a spinal manometer. However the reasons for non-adherence were not explored in this study but are likely to be similar to the findings of other studies.^18^

Only 23.4% of the patients admitted with CCM had ICP monitoring and therapeutic LPs. Although this adherence level is considerably lower than our target of 80%, it is higher than the compliance level of 14.9% reported from a study in a rural district hospital from KwaZulu-Natal in 2011.^16^ A review of our patients’ clinical records showed that clinicians preferentially prescribed analgesics for persistent headaches in patients with CCM rather than performing therapeutic LPs. However, such practices have been associated with worse neurological outcomes^18^ and there are concerns that the use of non-steroidal anti-inflammatory drugs (NSAIDS) could potentially increase the risk of amphotericin B-related nephrotoxicity.^22^ In fact, NSAIDS were prescribed as analgesics for 21.3% (27/127) of the patients during the induction phase with amphotericin B. Whilst therapeutic LPs remain ‘... the best form of “analgesia” for headaches associated with raised intracranial pressure...’^22^ (p. 18), the perceived onerous effort required for the consistent performance of serial LPs has been found to constitute a major factor limiting clinicians’ compliance with clinical guidelines on therapeutic LPs.^18^

Despite existing evidence on the dose-dependent nephrotoxic potential of amphotericin B,^23^ analysis of the pattern of amphotericin B prescription in this study showed limited evidence of weight-based dosing. In this study, in 43.6% of the cases, clinicians prescribed a 50 mg daily dose of amphotericin B. This may be because amphotericin B comes in standard preparations of 50 mg/vial in South Africa and this may thus represent a convenient dosing schedule for both doctors and nurses. Another possible factor limiting accurate weight-based dosing that was raised by those at the audit feedback meeting was the fact that some of the patients with CCM were too ill to get on a standard weighing scale. The reasons for this practice need to be explored in a further study. The retrospective nature of this study made it impossible to determine the impact of this practice on CSF sterilisation rate or the risk of amphotericin B-related nephrotoxicity.

Whilst amphotericin B remains the preferred and most effective drug for the treatment of serious systemic fungal infections such as CCM,^23, 24^ universal access to amphotericin B remains a challenge in developing countries.^8, 25^ In a retrospective case review series at a rural hospital in northern KwaZulu-Natal, only 35% of patients diagnosed with CCM received amphotericin B and only 8.1% completed the recommended 14 days of induction therapy.^16^ Apart from amphotericin B stock-outs, early mortality and renal impairment were the other major reasons why patients did not complete 14 days of induction therapy with amphotericin B in an audit in the Western Cape.^17^ In the study setting, drug stock-out did not appear to be a major problem; 92.1% of the patients received amphotericin B with only one case where the drug was not available. Deviations from the 14-day induction target were mainly a result of early mortality; clinicians’ decision to discontinue amphotericin B on account of improvement in the clinical status of the patient; and amphotericin B-related toxicity. Given the finding from a prospective study in India that even the standard 14-day amphotericin B monotherapy might be suboptimal for one third of patients,^26^ clinicians’ decision to discontinue amphotericin B before 14 days on the premise of clinical improvement remains a concern. It is pertinent to mention that the South African National Department of Health (NDOH) conceded that completing the 14-day course of amphotericin B might not always be ‘feasible’ given the high demands for hospital beds and thus recommended that ‘… an earlier switch to oral fluconazole may be considered … [*by the clinician*] … if there has been a good clinical response’^12^ (p. 169). The impact of this practice on CCM recurrence rates, CCM-related IRIS and survival outcomes warrants further study.

The prevailing guidelines during the study period (from the Southern African HIV Clinicians Society [SAHIVSOC] and the NDOH) included recommendations on the prevention, monitoring and management of amphotericin B-related toxicities.^10, 12^ The guidelines recommend pre-emptive saline loading and electrolyte (potassium and magnesium) supplementation; weekly haemoglobin monitoring; monitoring of potassium and creatinine levels; and management of ensuing electrolyte(s) and creatinine derangements.^10–12^ Similar to a clinical audit report from the Western Cape,^17^ clinicians’ adherence to these recommendations was suboptimal in the study setting. This was particularly poor with respect to prophylactic magnesium supplementation. These gaps have been attributed to inadequate awareness amongst clinicians regarding toxicity monitoring and prevention recommendations.^17^ Despite these gaps, the incidence of renal impairment was within the clinical audit target.

Optimal in-patient linkage to ART counselling services of patients diagnosed with CCM has been attributed to the integrated nature of district level services.^17^ All the patients whose HIV status was unknown or negative at admission for CCM were referred for counselling and testing. However, 22.5% (7/31) of the patients could not be counselled and/or tested mainly due to early mortality.

Similar to other reports from routine care in sub-Saharan Africa,^1, 16, 17, 27^ acute CCM survival outcomes were poor. The two-week in-patient mortality outcome of 55.9% (71/127) was worse than the preset audit target of 40%. Concerns about the persistently-high burden of CCM and the poor mortality outcome in routine care have led to calls for screening and treatment of early cryptococcal disease amongst patients with no prior CCM but with advanced immunodeficiency whenever they present for ART initiation.^22, 28^ This pre-emptive strategy, specifically targeting ART-naïve HIV-infected patients with a CD4 cell count < 100 cells/mm^3^, has been started in some provinces in South Africa.^29^ In 2013, the SAHIVSOC published an updated guideline that reflects recent recommendations for the prevention, diagnosis and management of CCM amongst HIV-infected patients.^22^

### Strengths and limitations

Given the retrospective nature of this clinical audit, it was impossible to standardise the quality of information documented in the patients’ folders and there were instances of missing or incomplete clinical records. These limitations notwithstanding, the clinical audit and the subsequent feedback session provided a forum for stakeholders to learn and also discuss their concerns with regard to CCM management in the hospital. It also ensured that the reasons for the identified practice gaps and the ensuing recommendations were based on collective feedback and agreement.

## Conclusion

This audit of the quality of acute management of CCM in routine care showed gaps in the management of this life-threatening illness, despite the availability of context-specific guidelines. The low adherence to practice guidelines represents an opportunity to improve care through feedback of the audit results coupled with implementation of a comprehensive action plan based on stakeholders’ recommendations. Thus, in conjunction with the Quality Improvement Manager at the hospital, the audit team has developed an action plan aimed at improving the management of CCM at the facility. A repeat audit will be conducted six months after the implementation of the plan in order to evaluate its impact and to ensure that improvements are sustained.
